# Moderate physical exercise improves lymphocyte function in melanoma-bearing mice on a high-fat diet

**DOI:** 10.1186/s12986-019-0394-z

**Published:** 2019-09-12

**Authors:** Cesar Miguel Momesso dos Santos, Vinicius Leonardo Sousa Diniz, André Luis Lacerda Bachi, Laiane Cristina dos Santos de Oliveira, Tamara Ghazal, Maria Elizabeth Pereira Passos, Heloisa Helena de Oliveira, Gilson Murata, Laureane Nunes Masi, Amanda Roque Martins, Adriana Cristina Levada-Pires, Rui Curi, Sandro Massao Hirabara, Donald F. Sellitti, Tania Cristina Pithon-Curi, Renata Gorjão

**Affiliations:** 1 Interdisciplinary Post-graduate Program in Health Sciences, Cruzeiro do Sul University, Rua Galvão Bueno, 868, CEP: 01506 000, Liberdade, São Paulo, Brazil; 2 Department of Otorrhynolaringology, Federal University of São Paulo, São Paulo, Brazil; 3 Brazilian Institute of Teaching and Research in Pulmonary and Exercise Immunology (IBEPIPE), São Paulo, Brazil; 4 Department of Physiology and Biophysics, Institute of Biomedical Sciences, University of São Paulo, Av. Prof. Lineu Prestes, 1374, CEP: 05508-900, Butanta, São Paulo, Brazil; 5 Department of Medicine, Uniformed Services University of Health Sciences, 4301 Jones Bridge Road, Bethesda, MD USA

**Keywords:** Cytokines, Th1 lymphocytes, B16F10 cells, Cancer, Obesity, Immune function

## Abstract

**Background:**

Obesity can lead to a chronic systemic inflammatory state that increases the risk of cancer development. Therefore, this study aimed to evaluate the alterations in tumor non-infiltrated lymphocytes function and melanoma growth in animals maintained on a high-fat diet and/or moderate physical exercise program in a murine model of melanoma.

**Methods:**

Female mice were randomly divided into eight groups: 1) normolipidic control (N), 2) normolipidic + melanoma (NM), 3) high-fat control (H), 4) high-fat + melanoma (HM), 5) normolipidic control + physical exercise (NE), 6) normolipidic melanoma + physical exercise (NEM), 7) high-fat control + physical exercise (HE), and 8) high-fat melanoma + physical exercise (HEM). After 8 weeks of diet treatment and/or moderate physical exercise protocol, melanoma was initiated by explanting B16F10 cells into one-half of the animals.

**Results:**

Animals fed a high-fat diet presented high-energy consumption (30%) and body weight gain (H and HE vs N and NE, 37%; HM and HEM vs NM and NEM, 73%, respectively), whether or not they carried melanoma explants. Although the tumor growth rate was higher in animals from the HM group than in animals from any other sedentary group, it was reduced by the addition of a physical exercise regimen. We also observed an increase in stimulated peripheral lymphocyte proliferation and a decrease in the T-helper 1 response in the HEM group.

**Conclusions:**

The results of the present study support the hypothesis that altering function of tumor non-infiltrated lymphocytes via exercise-related mechanisms can slow melanoma progression, indicating that the incorporation of a regular practice of moderate-intensity exercises can be a potential strategy for current therapeutic regimens in treating advanced melanoma.

## Background

Dietary intervention and regular physical exercise are principal behavioral changes that reduce the individual risk of developing certain cancers [1, 2]. Despite the association of dietary and activity-based behaviors with cancer incidence, the causal relationship between these behaviors and cancer induction and progression remains unresolved.

One possible mechanism underlying the association of diet with cancer development involves a direct relationship between increased total body adipose and high infiltration of inflammatory cells, such as T helper 1 (Th1) lymphocytes and M1 macrophages, into tissues prone to carcinogenesis [3–5]. Moreover, a decrease in T-regulatory (Treg) cells involved with the suppression of activated immune cells has been reported in obesity [6]. Recent studies have provided further support for the involvement of inflammatory pathways in melanoma tumor progression and have called particular attention to the generation of reactive oxygen species (ROS) and their role in tumorigenesis [7, 8].

The adipocyte-secreted hormone, leptin, stimulates Th1 proliferation and inhibits Treg cell function and, therefore, could be an important effector of obesity-based immune dysfunction, thereby increasing cancer risk [4]. Other mechanisms involve changes in the profile of infiltrated immune cells in adipose and tumor tissues that may contribute to tumor progression [9, 10].

The anti-tumor actions of individual cellular components of the immune system have been extensively documented and used as a base for new cancer treatments, including the class of drugs known as anti-programmed cell death protein 1 (PD-1) inhibitors [11, 12]. Treatments such as PD-1 inhibition and stimulation of antigen-specific cytotoxic responses to tumor cells can result in enhanced inflammation in the tumor tissue and a generally salutary response to treatment [13]. However, a balanced immune system response is essential for a positive outcome, because the beneficial effects of tumor inflammation may be countered and negated by the pro-tumorigenic effects of an uncontrolled inflammatory response favoring the growth of tumor cells, rather than their destruction. Some of these pro-tumorigenic signaling pathways of the inflammatory response can augment tumor progression via the activation of specific transcription factors, including signal transducers and activators of transcription STAT 3 and nuclear factor NF-kB [14], in tumor cells. STAT3 activation can promote increased expression of the anti-apoptotic protein Bcl-xL, which permits the survival and continued growth of tumor cells. NF-kB is the major activator of the immune response and inflammation. Its activation increases the expression of chemokines and inflammatory cytokines, thereby increasing leukocyte infiltration, growth factor secretion, and adhesion molecule expression to enhance both tumor angiogenesis and metastasis [15].

In addition to dietary changes that can alter cancer risk via changes in the immune system, the level of physical exercise can also reduce individual cancer risk by altering leukocyte response. Among the factors mediating the link between exercise and low cancer risk is the modulation of certain proinflammatory mediators, including tumor necrosis factor (TNF)-alpha, monocyte chemoattractant protein 1, plasminogen activator inhibitor-1, IκB kinase, and NF-kB, and other growth factors and hormones, including adiponectin and interleukin (IL)-10 [16]. Some authors [17, 18] have suggested that exercise training can promote an increase in metabolic activity, lysosomal enzyme activity, peritoneal macrophage phagocytic activity and also an increased cytotoxic capacity of natural killer (NK) cells. Momesso et al. [19] demonstrated that obesity promotes an imbalance in the immune system function characterized by elevated lymphocyte proliferation due to a decrease in the percentage of Treg cells, and these effects were partially reversed by moderate physical exercise.

Estimates from the National Cancer Institute [20] show that the number of new cases of melanoma in the United States was 22.2 per 100,000 inhabitants per year between 2012 and 2016. The number of deaths was 2.5 per 100,000 inhabitants per year. Data from 2014 indicate that 1169,351 people lived with melanoma in the United States. In 2019, it was estimated that there would be 96,480 new cases of skin melanoma and that 7230, people would die due to this disease in the United States. Multiple factors are involved in the progression of this cancer, ranging from the genetic background of the host and the genetic and phenotypic makeup of the tumor to epigenetic influences on tumor growth and its interactions with the surrounding microenvironment [21]. Modulation of the microenvironment by direct cell contact with the tumor or by secretion of proteins and exosomes [22] promotes reciprocal changes in the tumor phenotype, allowing the acquisition of aggressive features, such as uncontrolled proliferation, evasion from growth suppressors, escape from the immune response, induction of inflammation, angiogenesis, genomic instability, mutations, resistance to cell death, invasion, and metastasis [23].

During obesity development, physiological function of augmented adipose tissue is altered leading to increased adipokines secretion such as leptin and TNF-alpha. In studies performed in humans, it has been observed that mesenteric adipose tissue has high relationship with the metabolic complications related to obesity due to elevated level of inflammatory profile in this local in comparison to other tissues [24]. Mesenteric lympho nodes are the main lymphoid organs where occur lymphocyte proliferation, differentiation and dissemination and are susceptible to alterations in adipose tissue. The peripheral lymphocytes located in these organs can be differentially altered, favoring the inflammatory response in other tissues and facilitating the tumor growth. Therefore, the present study aimed to investigate the effects of high-fat diet and moderate physical exercise program on tumor non-infiltrated lymphocytes in C57BL-6 mice with melanoma.

## Methods

### Animal treatment

All experiments were approved by the Ethics Committee for Animal Experimentation of the Cruzeiro do Sul University and conducted according to experimental protocols approved in Brazil (Ethics Committee for Animal Experimentation 001/2014). All experiments were performed in accordance with relevant guidelines and regulations.

Female C57BL/6 mice (*n* = 80, 6 weeks of age) were purchased from the School of Medicine, University of Sao Paulo (Sao Paulo, Brazil). Five animals were housed (cage dimensions: 27 × 17 × 13 cm) in a controlled environment with a light-dark cycle of 12–12 h and temperature of 23 °C ± 2 °C. Previous studies showed that B16F10 murine melanoma growth and progression can occur equally both in male [25] and female [26–30] C57BL/6 mice. The protocol of the present study was based in the studies of Correa and colleagues [28] and Bachi et al. [26, 27], that maintained the use female C57BL/6 mice strain in the in vivo experiments.

Initially, the animals were divided into four groups: 1) normolipidic diet (N), 2) normolipidic diet + physical exercise protocol (NE), 3) high-fat diet (H), and high-fat diet + physical exercise protocol (HE). These regimens were continued for 8 weeks. Afterwards, B16F10 melanoma cells (1 × 10^5^ cells per 100 μL) were implanted in animals from each of the four groups. Sterile phosphate-buffered saline (PBS) equivalent to the volume of melanoma cells (melanoma control) was injected in control group. Therefore, the final experimental protocol comprised eight groups: 1) normolipidic control (N), 2) normolipidic + melanoma (NM), 3) high-fat control (H), 4) high-fat+ melanoma (HM), 5) normolipidic control + physical exercise (NE), 6) normolipidic melanoma + physical exercise (NEM), 7) high-fat control + physical exercise (HE), and 8) high-fat melanoma + physical exercise (HEM). The experiments were performed three times resulting in a final number of animals described in the figure legends.

For tumor growth analysis, mice were evaluated until the 31st day after melanoma induction. In relation to the other analysis, the animals were euthanized on the 21st day after melanoma induction, because until this time point all animals were alive.

Mice were divided into groups according to diets containing different amounts of lipids. The normolipidic diet contained 10% of energy from fat, whereas the high-fat diets provided 60% of energy from lipids. The detailed composition of the diets is shown in Table 1.

**Table 1:** Diet composition

Ingredients (g/kg)	N	H
Starch	465.7	115.5
Sucrose	100	100
Casein	140	200
Soybean oil	4	35
Lard	36	315
Energy	10.6	22.5

*N* normolipidic diet, *H* high-fat diet

### Melanoma induction

The melanoma induction was performed as described by Bachi et al. [27] and Correa et al. [28]. Subconfluent monolayers of B16F10 melanoma cells were harvested after trypsin treatment, counted, and resuspended in RPMI-1640 culture medium enriched with 2 mM glutamine, 24 mM sodium bicarbonate (NaH_2_CO_3_), 20 mM HEPES, 10% fetal bovine serum (FBS), and antibiotics (1000 U/mL penicillin and 1000 μg/mL streptomycin). B16F10 (1 × 10^5^ cells per 100 μL) were subcutaneously injected in the left hind limb of mice at the eighth week of diet treatment and/or physical exercise training. Animals were maintained under the same treatment as described before and checked daily for tumor development. Tumor growth was monitored three times per week, and the tumor volume was determined as follows: [maximum diameter × (minimum diameter)^2^]/2 [31]. Mice with a subcutaneous mass greater than 20 mm^3^ were considered positive for the presence of tumors.

### Exercise protocol

Animals were trained on a treadmill with individual lanes designed for small animals, without electrical stimulation (Insight, São Paulo, Brazil). The trained groups performed moderate physical exercise from the first week (6-week-old mice) until the tenth week of the experiment. Mice were subjected to treadmill adaptation three times a week, 10 min per day during the week before beginning the experiment. Animals were subjected to treadmill adaptation at a speed of 0.5 km/h. Afterwards, the animals were randomly assigned to trained and untrained groups.

The intensity of the exercise training was determined according to an acute incremental exercise test on the treadmill [32]. Briefly, the exercise intensity was increased by 3 m/min (beginning with 8 m/min) every 3 min, at 5% of inclination, until exhaustion (defined by the inability to maintain running speed). The maximal speed was used to calculate moderate physical exercise intensity corresponding to a percentage of 45–55% of maximal speed. The training regimen was conducted over 10 weeks, five times a week, 1 h per day, at 5% grade. This protocol comprised 10 min of warm-up at 45% of the maximum speed, followed by 20 min at 50% of maximum speed; 20 min at 55% of the maximum speed; and 10 min at 45% of the maximum speed. All animals were trained between 10:00 a.m. and 02:00 p.m.. Sedentary mice were submitted to similar stress condition as exercised animals. Therefore, mice were exposed to a stationary treadmill during the same period of daily training.

To evaluate the effectiveness of the training program, sedentary and exercised mice were subjected to the incremental load test [33] on the treadmill during the first, fourth, and eighth weeks of the study. This test provided the total distance, total time, and maximal speed run for each animal.

### Body weight and food intake

All animals were weighed on a digital scale every week throughout the experimental period. Animal body weight gain was calculated as follows: final body weight − initial body weight.

Food consumption was also measured throughout this period. For this purpose, known amounts of food were placed in each cage, and the remaining food pellets were weighed on a digital scale three times a week. Retroperitoneal and subcutaneous adipose tissues were collected and weighed at the end of the experimental protocol for calculating the final adiposity.

Lee index was calculated by cubic root of body weight in gram divided by naso-anal length in centimeters [34, 35].

### Muscle citrate synthase enzymatic activity

Soleus muscle samples (100 mg) were homogenized in 1 mL extraction buffer (pH 7.4) containing 50 mM tris-aminomethane and 1 mM ethylenediamine tetraacetic acid (EDTA). The tubes were kept on ice for 10 s, homogenized, and centrifuged (14,000×*g* for 1 min at 4 °C) for the separation of cellular debris. The supernatant was used for the analysis of citrate synthase enzymatic activity. TritonX-100 (0.05% v/v) was added to 1.0 mL of the final volume. Citrate synthase activity was assayed as described by Alp et al. [36].

### Serum leptin levels

Plasma leptin levels were determined using the mouse leptin ELISA Kit from ThermoFisher Scientific (Waltham, MA, USA), according to the manufacturer’s instructions. Plasma samples were diluted 1:20 to avoid exceeding the standard curve.

### Extraction of lymphocytes from mesenteric lymph nodes

Cells from mesenteric lymph nodes were extracted by pressing tissues against a steel mesh, as described by Ardawi and Newsholme [37]. Briefly, after collecting the lymph nodes, adipose tissue was carefully removed from the node to prevent contamination of the lymphatic tissue. The lymphoid tissues were then pressed into a stainless steel mesh in the presence of PBS (pH 7.4) to liberate the lymphocytes. The suspension containing PBS and lymphocytes was filtered through 70-μM nylon cell strainers and centrifuged at 250×*g* for 10 min at 4 °C.

### T-regulatory (Treg) and Th-17 cell counting

The percentage of Treg cells (CD4+, CD25+, and FoxP3+) was determined by incubating the lymphocytes (1 × 10^6^ cells) with anti-CD4 (PerCP-Cy5.5) and anti-CD25 (APC) antibodies for 30 min. Cells were fixed (1% formaldehyde in PBS) and incubated in BD Perm/Wash permeabilization buffer (Becton Dickinson, CA, USA) for 15 min at room temperature. Cells were washed and incubated with anti-FoxP3 antibody (Alexa Fluor 647), diluted 1:10, for 30 min. The negative control cells were incubated with the unreacted labeled IgG (Alexa Fluor 647 Rat IgG1Isotype; APC Rat IgG1 Isotype; PerCP-Cy5.5 Rat IgG1). A total of 20,000 CD4+ cells were loaded into a BD Accuri flow cytometer (Becton Dickinson); the resulting histograms were analyzed using the “BD CSampler Software” (Becton Dickinson).

The percentage of Th17 cells was determined by incubating isolated lymphocytes for 12 h with phorbol myristate acetate (PMA, 300 ng/mL) and ionomycin (1 μg/mL) in 800 μL RPMI 1640 medium supplemented with 10% FBS. CD4/IL-17A-positive lymphocytes were identified by sequential staining using specific antibodies. Thus, cells were incubated with anti-CD4 and subsequently in buffer BD Perm/Wash (Becton Dickinson,) for 15 min, followed by incubation with anti-IL-17A antibody PE (1:10) for 30 min, protected from light (PE Rat IgG1). Subsequently, cells were evaluated in a flow cytometer as described above.

### Cytokine production by stimulated lymphocytes in vitro

Lymphocytes were cultured in RPMI-1640 containing 2 mM glutamine, 24 mM NaH_2_CO_3_, 20 mM HEPES, 10% FBS, and antibiotics (1000 U/mL penicillin and 1000 μg/mL streptomycin) at 37 °C with 5% CO_2_ and 95% atmospheric air. First, lymphocytes (1 × 10^6^ cells) were incubated with PMA (300 ng/mL) and ionomycin (1 μg/mL) in 500 μL medium for 24 h. Afterwards, cells were centrifuged, and the collected supernatant was frozen at − 80 °C. TNF-alpha, IL-17, IL-4, IL-2, IL-10, and interferon (IFN)-gamma concentrations in the supernatant were determined by Cytometric Bead Array (CBA) using the BD™ CBA Th1Th2Th17 Mouse Cytokine Kit (BD Biosciences) and the BD Accuri flow cytometer. The results of CBA experiments were obtained with FCAP Array software (v 3.0). The limit of detection was 0.1 pg/mL for IL-2, 0.03 pg/mL for IL-4, 1.4 pg/mL for IL-6, 0.5 pg/mL for IFN-gamma, 0.9 pg/mL for TNF-alpha, 0.8 pg/mL for IL-17A, and 16.8 pg/mL for IL-10.

### Lymphocyte proliferation

The proliferative capacity was assessed by bromodeoxyuridine (BrdU) incorporation into the DNA of cells using the APC BrdU Flow Kit (BD Biosciences). Lymphocytes (1 × 10^6^) were resuspended in 1 mL RPMI-1640 supplemented with 10% FBS containing antibiotics (10,000 U penicillin, 10 mg/L of streptomycin). After centrifugation, lymphocytes were cultured in 96-well plates containing 5 × 10^5^ cells per well and incubated at 37 °C in an atmosphere of 95% air and 5% CO_2_. At the beginning of culture, lymphocytes were stimulated with concanavalin A (5 μg/mL) and incubated with 10 μM BrdU for 48 h. After this period, cells were permeabilized and fixed with BD Cytofix/Cytoperm Buffer (100 μL of 1 × 10^6^ cells at room temperature for 30 min). Afterwards, cells were washed with 500 μL of BD Perm/Wash buffer and incubated with 100 μL of DNAse (300 mg/mL in DPBS) for 1 h at 37 °C. Cells were washed with 500 μL of the BD Perm/Wash Buffer and incubated with 50 μL of BD Perm/Wash buffer containing anti-BrdU antibody conjugated to APC (1:50) for 20 min at room temperature. Cells were washed again as described above and resuspended in 200 μL of PBS (1% bovine serum albumin). Finally, the cells were analyzed by flow cytometry using the BD Accuri (Becton Dickinson). Ten thousand events were acquired per sample in the histograms. The histograms were analyzed using the “BD-C6 Sampler Software” (Becton Dickinson). The results were shown as mean fluorescence intensity. These data indicate that cells with a higher proliferation rate have a higher fluorescence intensity.

### Statistical analysis

Results were expressed as the arithmetic mean ± standard error of the mean. For statistical analysis of the results, a two-way ANOVA was performed followed by Bonferroni’s post-test. For results of tumor growth analysis, a two-way repeated measurements ANOVA was performed for multiple comparisons using GraphPad Prism, version 6 for Windows (GraphPad). A *p* value ≤0.05 was considered statistically significant.

## Results

### Body and adipose tissue weight and food intake

Table 2 lists the mean body weight and Lee index for the eight experimental groups at the end of the experimental period. Although the naso-caudal length did not significantly differ among groups, significant differences in the final body weight were found on the basis of dietary fat intake and exercise.

**Table 2:** Characterization of tumor-bearing mice maintained on a high-fat diet and moderate physical exercise

	N	NE	H	HE	NM	NEM	HM	HEM
Weight gain (g)•	6.2 ± 0.59	4.28 ± 0.50	11.0 ± 1.0*	9.17 ± 1.34*^$ #^	4.03 ± 0.38	3.47 ± 0.28	12.27 ± 1.55^&^	11.41 ± 1.28^&¶^
Nasal-caudal lengh (cm)	17.38 ± 0.09	17.23 ± 0.14	16.9 ± 0.36	16.8 ± 0.2	17.32 ± 0.13	17.26 ± 0.15	17.22 ± 0.09	17.40 ± 0.1
Lee Index (g/cm^3^)	1.62 ± 0.02	1.57 ± 0.02	1.78 ± 0.02	1.67 ± 0.03	1.62 ± 0.01	1.60 ± 0.0	1.71 ± 0.41	1.73 ± 0.02
Adipose Tissue♦	0.09 ± 0.05	0.07 ± 0.06	0.17 ± 0.08*	0.15 ± 0.03*^$^	0.07 ± 0.05	0.03 ± 0.02	0.18 ± 0,1^&^	0.17 ± 0.12^&¶^

•Weight gain was calculated as follows: final weight − initial weight

**♦**Adipose tissue values were calculated as follows: (retroperitoneal + subcutaneous tissue)/initial weight. *P < 0.05 vs N. ^&^*P* < 0.05 vs NM. ^#^*P* < 0.05 vs H. ^$^*P* < 0.05 vs NE. ^¶^*P* < 0.05 vs NEM. N = 9, NM = 8, H = 8, HM = 9, NE = 8, NEM = 8, HE = 8, HEM = 8

Normolipidic control (N); Normolipidic + melanoma (NM); High-fat control (H); High-fat + melanoma (HM); Normolipidic control + physical exercise (NE); Normolipidic melanoma + physical exercise (NEM); High-fat control + physical exercise (HE); High-fat melanoma + physical exercise (HEM)

Mice that received a high-fat diet (without a tumor burden) gained nearly twice as much weight as those maintained on a balanced diet (6.2 vs 11.0 g, respectively), whereas exercise significantly reduced the weight gain in animals fed a high-fat diet compared with that in the non-exercised groups. The tumor-bearing animals also gained a significant amount of weight induced by the high-fat diet, and this effect was unaffected by exercise. The total adipose tissue in each group also reflected the dietary regimen, but exercise did not affect this parameter in either the tumor-free or the tumor-bearing groups.

### Physical performance and soleus muscle citrate synthase activity

**Fig. 1:**
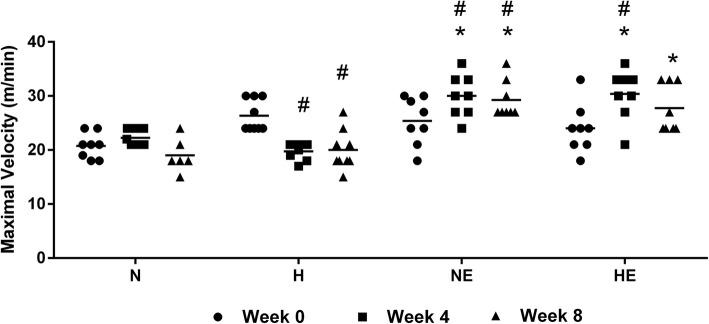
Evaluation of physical performance in mice maintained on either a high-fat or normolipidic diet and subjected to moderate physical exercise or allowed to remain sedentary. C57BL6 mice were maintained on a diet containing either 60% or 10% of energy from lipids and were either trained on a treadmill at moderate intensity for 8 weeks or were allowed to remain sedentary. Sedentary and exercised mice were subjected to the incremental load test on the treadmill during the first (week 0), fourth, and eighth weeks of the study. Subsequently, B16F10 tumor cells were injected, and physical performance was measured as described in the Methods. Data are presented as mean ± standard error of the mean. *P < 0.05 vs sedentary group. ^#^*P* < 0.05 vs week 0. N group, *n* = 8; H group, *n* = 9; NE group, *n* = 8 and HE group, *n* = 9. Normolipidic control (N); High-fat control (H); Normolipidic control + physical exercise (NE); High-fat control + physical exercise (HE)

In order to ascertain the adaptation of animals to aerobic physical exercise training, we measured soleus muscle citrate synthase activity in each of the four diet/exercise groups without a tumor burden over the course of 8 weeks (Table 3). There was a significant time-dependent increase in citrate synthase activity with continuous physical exercise in mice fed a balanced diet or a high-fat diet, suggesting increased respiratory activity in the muscle tissue of these animals (Table 3). The melanoma-bearing groups also showed increased citrate synthase activity with exercise, but this increase was only significant in the mice maintained on a high-fat diet. Animals subjected to exercise training also showed a high physical performance (Normolipidic control vs Normolipidic control + physical exercise, 35%; High-fat control vs High-fat control + physical exercise 26%, respectively), as shown in Fig. 1.

**Table 3:** Citrate synthase activity in soleus muscle of mice maintained on a high-fat diet and moderate physical exercise

	N	NE	H	HE	NM	NEM	HM	HEM
CS activity [nmol.(min.mg protein)]	0.95 ± 0.03	1.21 ± 0.10*	1.0 ± 0.04	1.20 ± 0.06^#^	0.98 ± 0.02	1.07 ± 0.04	0.87 ± 0.04	1.08 ± 0.03^&^

CS: citrate synthase. *P < 0.05 vs N. ^&^*P* < 0.05 vs HM. ^#^*P* < 0.05 vs H. N = 9, NM = 8, H = 8, HM = 9, NE = 8, NEM = 8, HE = 8, HEM = 8

Normolipidic control (N); Normolipidic + melanoma (NM); High-fat control (H); High-fat + melanoma (HM); Normolipidic control + physical exercise (NE); Normolipidic melanoma + physical exercise (NEM); High-fat control + physical exercise (HE); High-fat melanoma + physical exercise (HEM)

### Melanoma growth

**Fig. 2:**
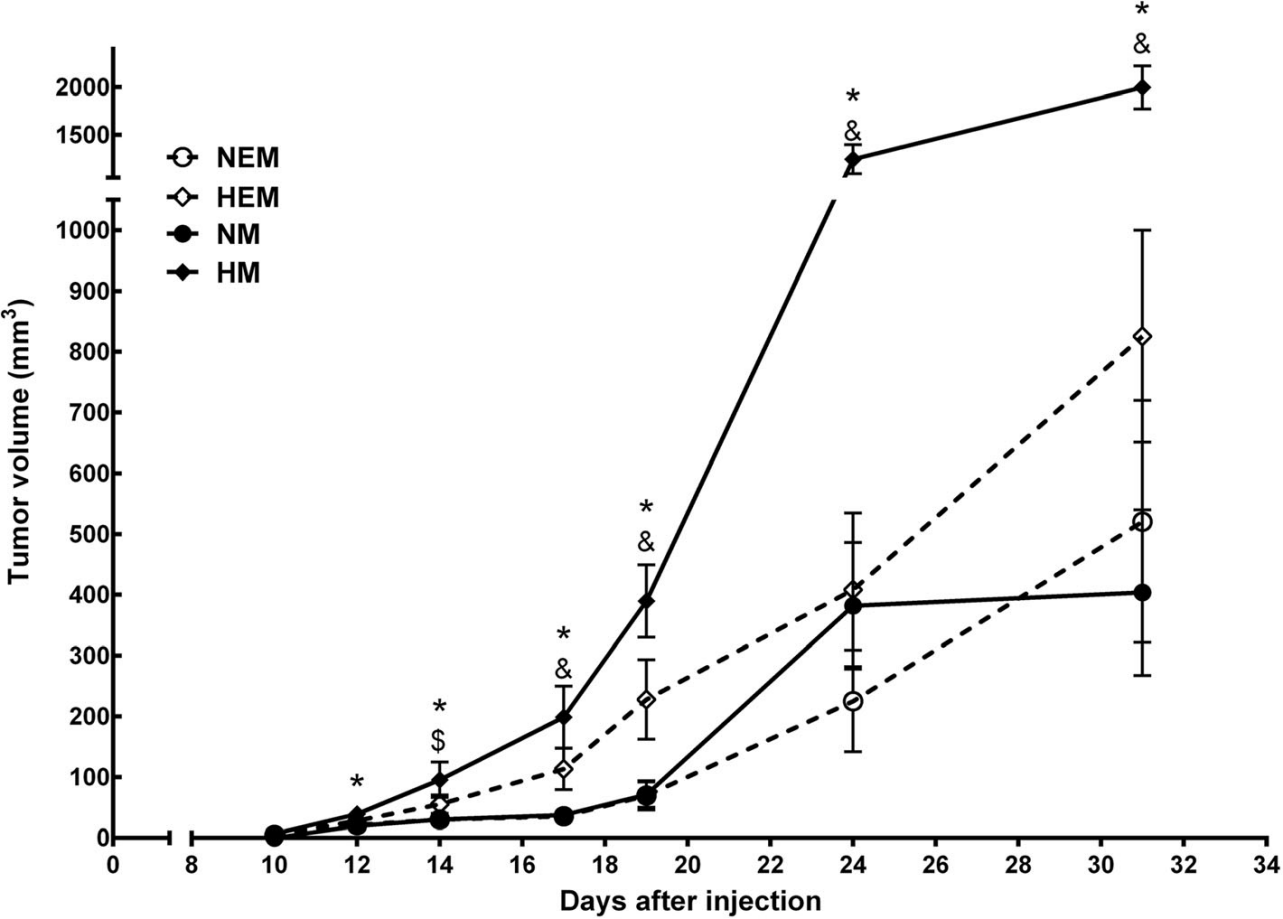
Effect of a high-fat diet and moderate physical exercise on tumor growth after injection of B16F10 tumor cells. C57BL6 mice were maintained on a diet containing either 60% or 10% of energy from lipids and were either trained on a treadmill at moderate intensity for 8 weeks or left untrained. Subsequently, B16F10 tumor cells were injected, and the tumor growth was recorded for 4 weeks as described in the Methods. Data are presented as mean ± standard error of the mean. *P < 0.05 NM versus HM groups. ^$^*P* < 0.05 NEM versus HEM; ^&^*P* < 0.05 between HM versus HEM. NM group, *n* = 8; HM group, *n* = 9; NEM group, *n* = 8 and HEM group, *n* = 9. Normolipidic control (N); Normolipidic + melanoma (NM); High-fat control (H); High-fat + melanoma (HM); Normolipidic control + physical exercise (NE); Normolipidic melanoma + physical exercise (NEM); High-fat control + physical exercise (HE); High-fat melanoma + physical exercise (HEM)

To gauge the contribution of diet and/or physical exercise to overall melanoma growth, we measured the tumor volume in the four tumor-bearing subgroups from day 10 to day 31 (Fig. 2). The high-fat diet significantly increased tumor growth by approximately twice the growth rate in the mice fed a balanced diet. The elevation of the tumor growth rate induced by the high-fat diet was reduced by the continuous exercise regimen.

### T lymphocyte percentage

**Fig. 3:**
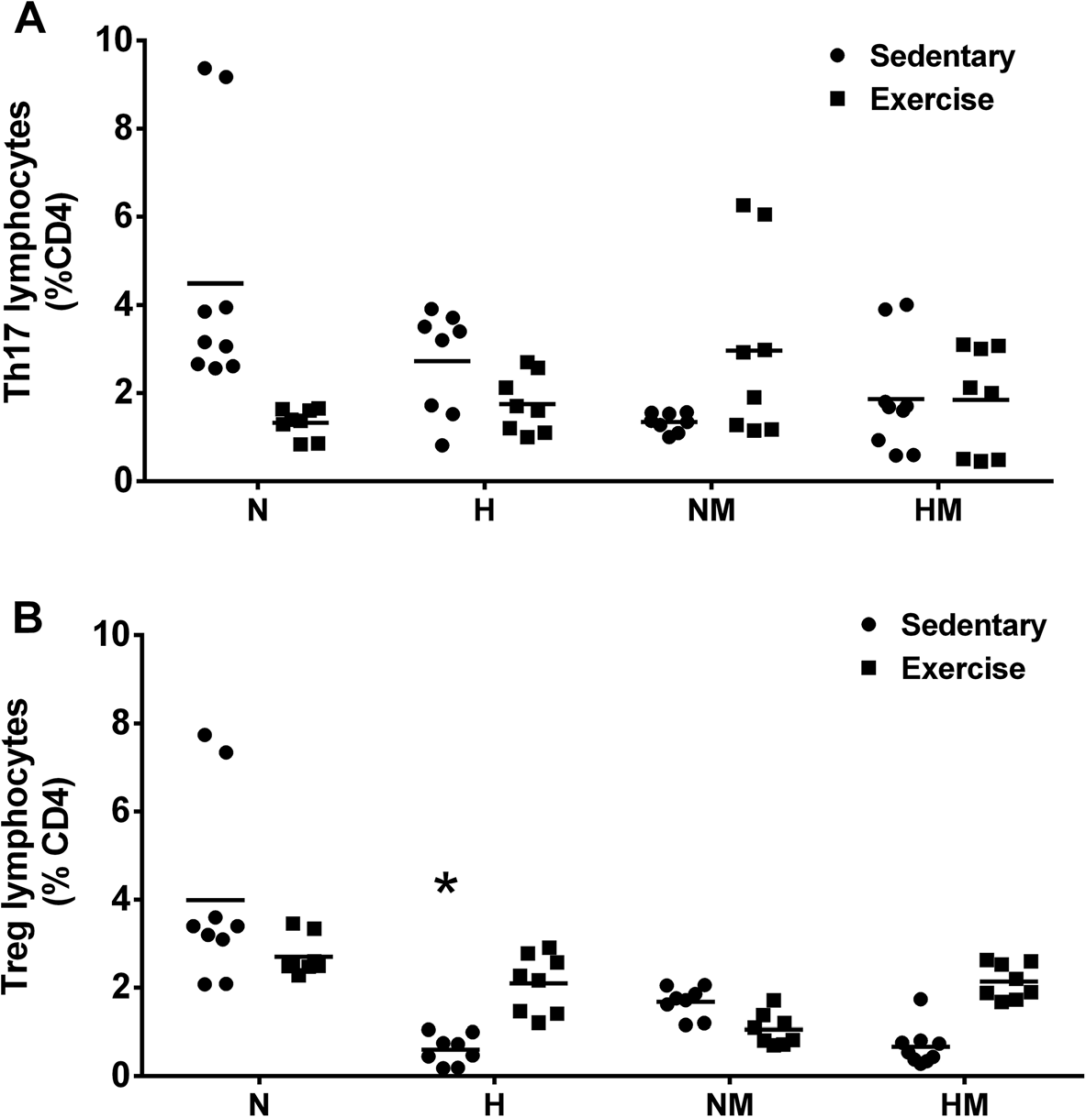
Effect of a high-fat diet and moderate physical exercise on the Th17 (**a**) and Treg (**b**) fractions of total CD4+ lymphocytes in lymph nodes of tumor-bearing mice. C57BL6 mice were maintained on a diet containing either 60% or 10% of energy from lipids and were either trained on a treadmill at moderate intensity for 8 weeks or left untrained. Subsequently, B16F10 tumor cells were injected, and animals were evaluated for 4 weeks. T regulatory cells were identified by labeling with anti-CD4 and anti-Foxp3. Th17 cells were identified by labeling with anti-CD4 and anti-IL-17. Data are presented as mean ± standard error of the mean, where, *P < 0.05 versus N. *N* = 9, NM = 8, H = 8, HM = 9, NE = 8, NEM = 8, HE = 8, HEM = 8. Normolipidic control (N); Normolipidic + melanoma (NM); High-fat control (H); High-fat + melanoma (HM); Normolipidic control + physical exercise (NE); Normolipidic melanoma + physical exercise (NEM); High-fat control + physical exercise (HE); High-fat melanoma + physical exercise (HEM)

Because Th-17 cells are known to mediate inflammatory changes [38] and inhibit tumor growth in mice via secretion of specific cytokines [39], we investigated the relative abundance of this T-helper cell subset in the mesenteric lymph nodes to determine a possible correlation between Th-17 cell number and either diet or exercise or both (Fig. 3). The percentage of Th17-positive cells did not significantly differ among the groups, indicating that neither diet nor exercise in the presence of tumor affected the proportion of this T-cell population in the mesenteric lymph nodes (Fig. 3a).

In contrast, the percentage of Treg lymphocytes was significantly reduced in sedentary animals maintained on high-fat diets compared with that in animals receiving balanced diets, supporting previously published findings about a negative association between Treg and adiposity (Fig. 3b). Moreover, in the exercised group, the percentage of Treg cells was similar between the high-fat diet group and balanced diet group. Therefore, exercise appeared to attenuate the reduction in Treg cells from the high-fat diet sedentary group in comparison to the control group. However, melanoma-bearing mice exhibited no difference in the percentage of Treg cells based on either diet or exercise.

### Lymphocyte proliferation

**Fig. 4:**
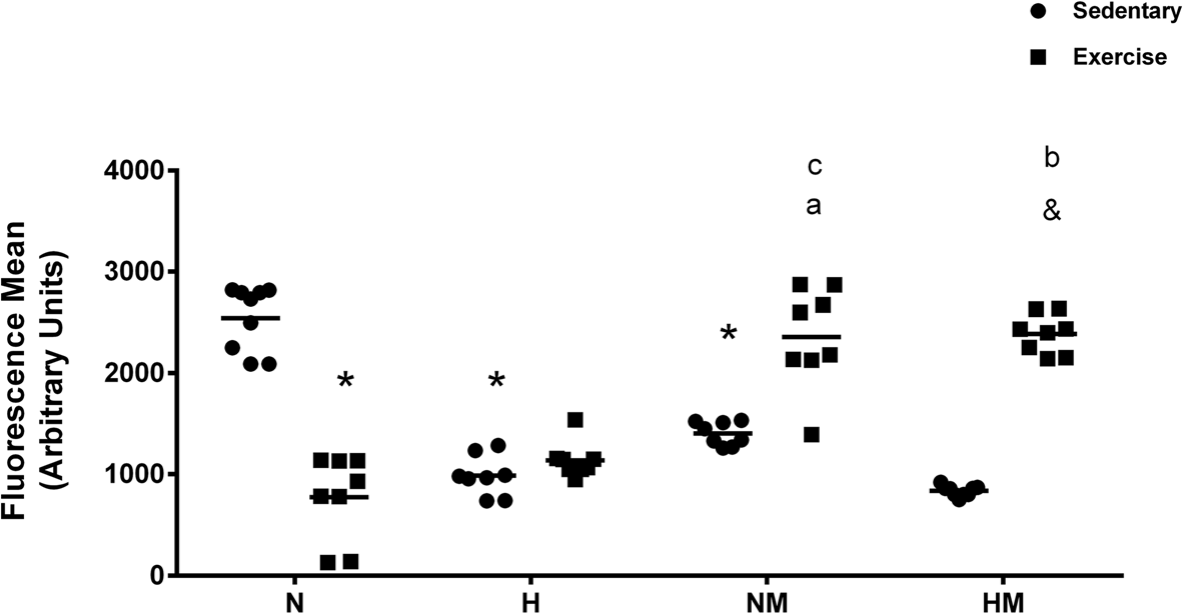
Effect of a high-fat diet and moderate physical exercise on lymphocyte proliferation in tumor-bearing mice. C57BL6 mice were maintained on a diet containing either 60% or 10% of energy from lipids and were either trained on a treadmill at moderate intensity for 8 weeks or left untrained. Subsequently, B16F10 tumor cells were injected, and animals were evaluated for 4 weeks for lymphocyte proliferation as described in the Methods. Data are presented as mean ± standard error of the mean. *P < 0.05 versus N sedentary; ^&^P < 0.05 versus HM sedentary; ^a^P < 0.05 versus NM sedentary; ^b^ *P* < 0.05 versus H exercise; ^c^*P* < 0.05 versus N exercise. *N* = 9, NM = 8, H = 8, HM = 9, NE = 8, NEM = 8, HE = 8, HEM = 8. Normolipidic control (N); Normolipidic + melanoma (NM); High-fat control (H); High-fat + melanoma (HM); Normolipidic control + physical exercise (NE); Normolipidic melanoma + physical exercise (NEM); High-fat control + physical exercise (HE); High-fat melanoma + physical exercise (HEM)

To gain insight into how exercise and dietary fat intake might affect immune function via changes in functional T-cell populations, we assayed lymphocyte proliferation in mesenteric lymph nodes in each of the eight groups (Fig. 4). Exercise significantly reduced lymphocyte proliferation in the absence of melanoma compared with that in the sedentary normolipidic control (N). Moreover, the high-fat diet (H) decreased lymphocyte proliferation in comparison to the balanced diet in sedentary animals without tumor burden. The presence of tumor, decreased proliferative capacity of lymphocytes in normolipidic groups. Lymphocyte proliferation is higher in exercised mice from melanoma group in comparison with animals without tumor burden. However, exercise increased lymphocyte proliferation in melanoma-bearing mice compared with that in the sedentary group, regardless of the dietary regimen administered to the animals (Fig. 4).

### Serum leptin

**Fig. 5:**
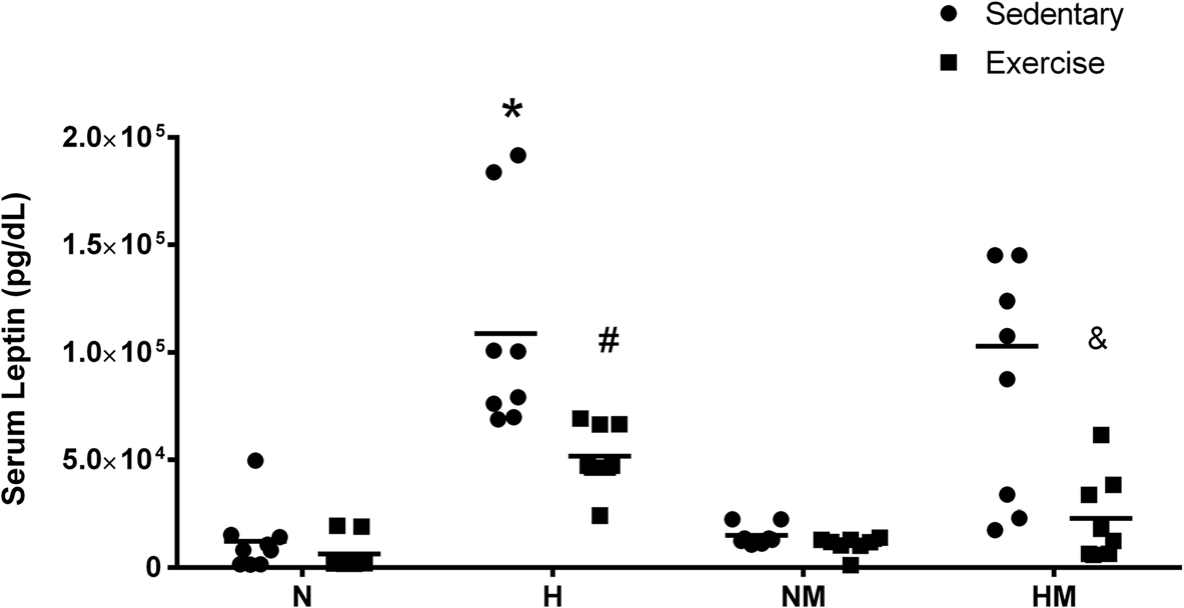
Serum leptin levels in mice with and without melanoma, and with or without high-fat diet and moderate physical exercise. C57BL6 mice were maintained on a diet containing either 60% or 10% of energy from lipids and were either trained on a treadmill at moderate intensity for 8 weeks or left untrained. Subsequently, B16F10 tumor cells were injected, and serum levels of leptin were measured for 4 weeks as described in the Methods. Data are presented as mean ± standard error of the mean, where, *P < 0.05 versus N; ^#^P < 0.05 versus H sedentary; ^&^*P* < 0.05 versus HM sedentary. N = 9, NM = 8, H = 8, HM = 9, NE = 8, NEM = 8, HE = 8, HEM = 8. Normolipidic control (N); Normolipidic + melanoma (NM); High-fat control (H); High-fat + melanoma (HM); Normolipidic control + physical exercise (NE); Normolipidic melanoma + physical exercise (NEM); High-fat control + physical exercise (HE); High-fat melanoma + physical exercise (HEM)

Because specific signaling molecules governing either adiposity or inflammation could underlie the changes in lymphocyte proliferation and profile observed in response to exercise and/or diet described above, we assayed the serum levels of leptin. There was a marked elevation in the serum levels of leptin in animals fed a high-fat diet (H) compared with those in the control group (N) and a significant reduction induced by continuous exercise in both the tumor-bearing and tumor-free (H) mice (Fig. 5). The serum levels of leptin were, therefore, associated with the estimated amount of adipose tissue (Table 1).

### Cytokine secretion by lymphocytes

**Fig. 6:**
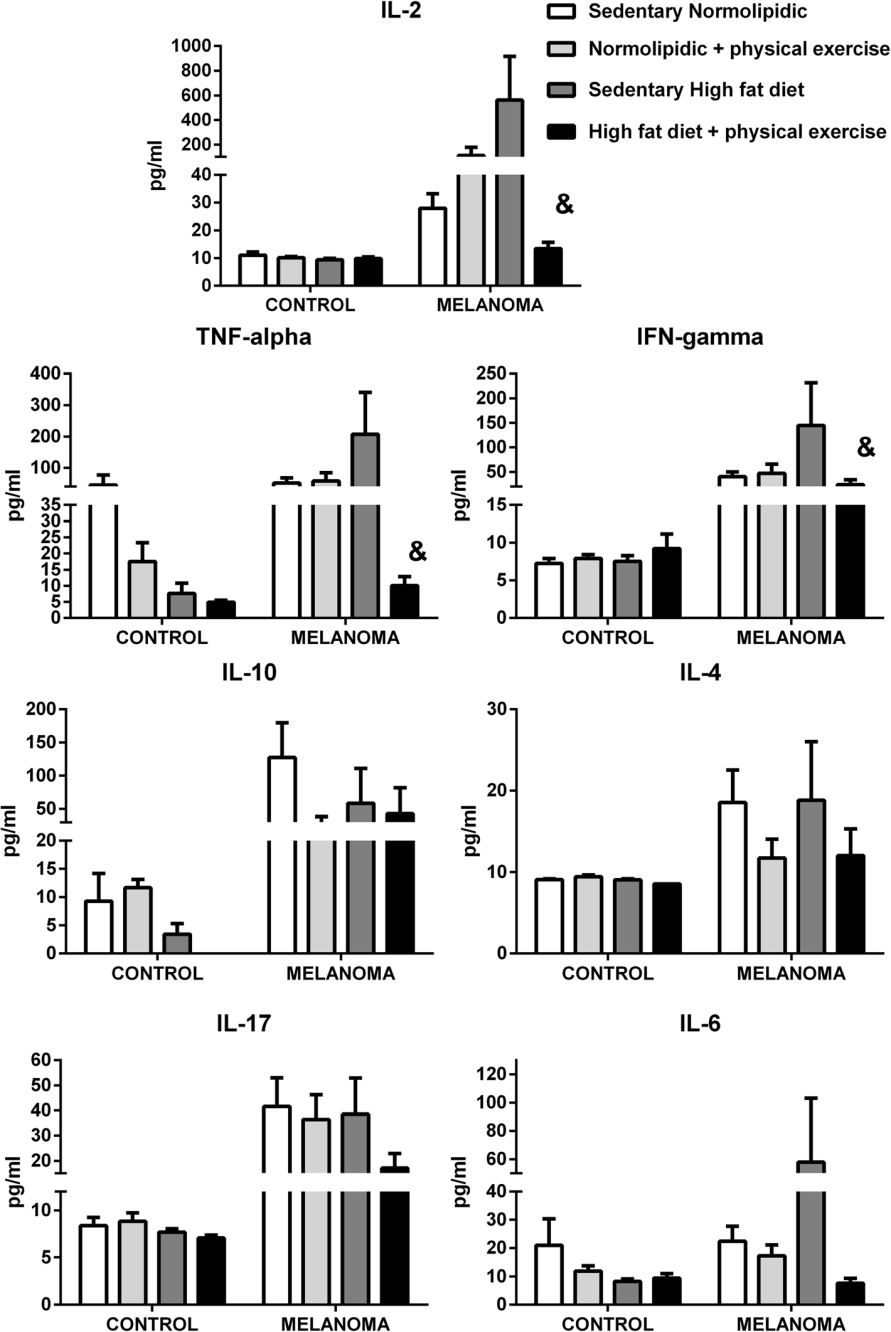
Cytokine levels in the supernatant from cultures of lymph nodes lymphocytes isolated from mice with and without melanoma, either with or without a high-fat diet and moderate physical exercise. C57BL6 mice were maintained on a diet containing 60% or 10% of energy from lipids and were either trained on a treadmill at moderate intensity for 8 weeks or left untrained. Subsequently, B16F10 tumor cells were injected, and cytokine secretion in extracted lymphocytes was measure as described in the Methods. Data are presented as mean ± standard error of the mean, where, ^&^*P* < 0.05 vs. sedentary high-fat diet melanoma. N = 9, NM = 8, H = 8, HM = 9, NE = 8, NEM = 8, HE = 8, HEM = 8

IL-2, IFN-gamma and TNF-alpha secretions were significantly reduced by continuous exercise in the tumor-bearing mice fed a high-fat diet (Fig. 6). There were no significant differences in IL-4, IL-10, IL-17, and IL-6 secretions by lymphocytes in either the control or tumor-bearing mice receiving either the balanced diet or high-fat diet maintained in either a sedentary or exercised condition.

## Discussion

An imbalance in immune function has been noted in both overweight/obese subjects and animal models of obesity [40, 41]. Moreover, there is significant evidence indicating that regular moderate physical exercise can have a positive influence on this imbalance by improving the immune response and thus inhibiting early tumor development. In fact, Pedersen et al. [42] observed that physical exercise attenuated melanoma growth in normal-weight mice. Interestingly, McQuade et al. [43] found that obesity was predictive of improved response to therapy in male patients with melanoma. To the best of our knowledge, our study was the first to show the impact of moderate physical exercise on tumor growth and tumor non-infiltrated lymphocytes functions in obese mice.

Although the exercise regimen did not statistically change the weight of adipose tissue on comparing the HM and HEM groups, it was able to reduce tumor growth. Physiological changes promoted by physical exercise may have contributed to the reduced tumor growth. There is a clear positive association between obesity and the growth of melanoma cells in mice and a negative effect of exercise on the elevated rate of tumor growth.

In fact, obese animals submitted to exercise had tumor growth rates that were indistinguishable from those in sedentary mice receiving normolipidic diets (Fig. 2). Extrapolating this data to the clinical setting, we suggest, based on the present results, that the practice of moderate physical exercise throughout life can contribute to a slow tumor progression arising in obese individuals and thus allow the immune system to mount an effective response against the malignancy. Jones et al. [44] described, in a human breast cancer model, that the amount of voluntary exercise reduces the rate of tumor growth.

Epidemiological, clinical and experimental studies have shown that over nutrition leading to adipose tissue expansion increases the risk of malignant melanoma (MM) and worsens its prognosis [1–8], [45]. Accordantly, it has been observed that adipose cells can interact dynamically with cancer cells and induce cancer cell growth, invasion and metastasis [46, 47]. In addition, following the contact with cancer cells, mature adipocytes undergo differentiation or apoptosis, which in turn activates pro inflammatory cytokine secretion both around and within the tumor microenvironment favoring the melanoma growth.

Moreover, our purpose was to observe mesenteric lymph node lymphocyte profile in these different models of melanoma tumor: mice submitted to physical exercise and/or high fat diet. Therefore, other important mechanisms can be modulated by physical exercise and influence tumor growth. Likewise, studies conducted on an animal model of prostate cancer (Copenhagen or nude mice) reported that moderate exercise reduces tumor hypoxia, promoting the generation of ROS and leading to a less aggressive tumor phenotype [48]. In addition to increased ROS, other possible mechanisms underlying the beneficial effect of exercise in slowing tumor growth include the reduction in inflammatory mediators such as IFN-gamma and leptin.

Leptin is a satiety hormone acting in the hypothalamus to reduce hunger [49], but other studies have described pro-inflammatory, anti-apoptotic, and pro-angiogenesis actions of this hormone, indicating that it plays an important role in the stimulation of tumor growth [4, 5]. In our study, the serum levels of leptin were increased in mice fed a high-fat diet; this is one possible explanation for the increased growth rate of melanomas explanted into mice in this cohort. Physical exercise reduced the serum levels of leptin in both melanoma-free and tumor-bearing mice, as shown in Fig. 5.

Based on the study of Lord and colleagues [50] and the revision of Faggioni et al. [3] leptin has a potent action both in the innate and adaptative immune responses. Firstly, it is important to mention that the leptin receptor belongs to the class I of cytokine receptor family, which includes common signal-transducing components with IL-6-related family of cytokines such as IL-6, IL-11, LIF, CNTF, and oncostatin M. In special, it has been demonstrated that the leptin action on lymphocytes includes: 1) enhance in the proliferative response of human peripheral blood lymphocytes by acting on naive T lymphocytes; 2) increase in the expression of activation markers CD69, CD25, and CD71 in CD4+ and CD8+ cells; 3) regulation of cytokine production by T lymphocytes; 4) polarization of the T helper (Th) cells towards a Th1 phenotype by enhancing proliferation and IL-2 production of naive T cells; 5) raise in the IFN-gamma and decrease in the IL-4 production in memory CD4+ T cells; and 6) up-regulation of the expression of adhesion molecules, such as VLA-2 and ICAM-1, on CD4+ T cells.

More specifically, human Treg cells express leptin and its receptor (LepR), being highly expressed in freshly isolated Treg cells. It seems to be responsible for the in vitro anergic phenomena observed in this cellular subset. It was demonstrated that leptin receptor-deficient (db/db) mice display marked increase in the number and suppressive function of Treg cells. Treg lymphocytes are also associated with immunological tolerance due to their ability in inhibiting the activity of other immune cells. Changes in the percentage and function of these cells are associated with tumor progression [51]. In our study, we explored an important association between serum leptin levels and tumor non-infiltrated lymphocyte function based on the fact that serum levels of leptin were increased in mice fed a high-fat diet. Furthermore, we speculate that the decreased serum levels of leptin occurring with the moderate exercise program could play a role in reducing the inflammatory response in obese mice because leptin, acting via specific receptors, has been previously shown to downregulate the expansion of Treg cells, while exerting opposite effect on lymphocytes involved in inflammation [52]. These earlier observations are consistent with the inverse effect of exercise on leptin levels and the percentage of Treg cells that we also observed in the mice lacking melanoma. Treatment with the high-fat diet led to a low percentage of Treg lymphocytes compared with that in the group fed the normolipidic diet, and exercise in the high-fat group mantained the Treg value toward a similar value from that of balanced diet-fed group (Fig. 3). The significant effect of exercise on Treg concentration in the high-fat fed group, however, was not observed in the tumor-bearing animals (Fig. 3). Therefore, the decrease in leptin levels in exercised obese mice may suppress inflammation that would otherwise permit the occurrence of a critical initial phase of tumor development. However, future studies investigating leptin signaling in lymphocytes by using leptin receptor knockout mice will be important to evidence this relationship in melanoma models.

Mice fed a high-fat diet that participated in physical exercise training (HEM) had a low production of IL-2 and IFN-gamma (13.4 ± 2.2 and 24.0 ± 9.7 pg/mL, respectively) compared with that in the group fed the high-fat diet without exercise (563.3 ± 353.2 and 144.7 ± 87.0 pg/mL, respectively). Th1 cells, are characterized by secretion of IL-2, IFN-gamma and TNF-alpha, that are inflammatory cytokines related to recruitment of other leukocytes [53]. The data of present study suggest that Th1 immunity, is markedly reduced in melanoma-bearing animals fed a high-fat diet and engaged with physical exercise. In regard to a possible mechanism for the exercise effect, Kawanishi et al. [54] reported that physical exercise contributes to the inhibition of inflammation through the negative regulation of Toll-like receptor-4, and this effect could offer, at least in part, an explanation of the effect of exercise on these inflammatory cytokines.

Although Th1 immune response is associated with better antitumor response, the reduced levels of the cytokines related to this type of immune response found in the HEM group did not show impairment of the capacity of physical exercise decrease the tumor growth. Regarding the reduction of Th1 cytokines observed in HEM group, Jovicic and collaborators [55] showed that C57BL/6 mice treated with high-fat diet presented higher serum levels of IL-6, IL-13 and TGF-β than the normal diet group. In addition, the chronic strenuous-exercise increased basal glucocorticoid levels as well as catecholamines levels during exercise, which could favor Th2 immune response. These results provide evidence that these stress hormones released during exercise can selectively amplify Th2 responses while suppressing Th1. In fact, Dunn et al. [56] described tumor growth occurring in several different phases: elimination, balance, and escape. In the elimination phase, infiltrating immune cells into the tumor microenvironment play a role in immunological surveillance to identify and eliminate malignant and/or pre-malignant immunogenic cells recognized as foreign due to their transformation. In the balance phase, the immune cells continue to be effective in immune surveillance and the destruction of most malignant cells. However, there is an essentially “Darwinian” selection of tumor cells in which transformed cells become resistant to immunologic action and proliferate to form expanding tumor clones. In the escape phase, cells from these resistant clones are able to fully escape from the immune system due changes occurring in the immune system itself, involving altered cytokine levels and changes in specific T-cell ratios. In our study, the phase of melanoma tumor is characterized by an inflammatory status that is typical of the escape phase and tumor progression. Therefore, the decrease of inflammatory cells and mediators may be beneficial to a slowly tumor growth. This effect was promoted by physical exercise.

Furthermore, in relation to the findings that exercise-training was able to reduce the tumor growth in HEM group, Hojman and collaborators [57] reported that the control of cancer progression can be achieved by exercise training through direct effects on tumor intrinsic factors (growth rate, metastasis, tumor metabolism, and immunogenicity), or by regulating tumor growth through interplay with systemic factors, among other effects. By the way, intratumoral signaling networks are highly modifiable and modulated by numerous extrinsic factors which can be affected by exercise, such as increase in blood flow, shear stress, pH regulation, heat production, and sympathetic activation and endocrine effects (for instance, stress hormones, myokines, and circulating exosomes).

In this respect, in 2016, Pedersen and collaborators [42] showed that voluntary wheel running was able to decrease tumor growth through an exercise-dependent mobilization and redistribution of cytotoxic immune cells, as CD8+ and natural killer (NK) cells. These mobilization and redistribution of the immune cells occurs through mechanisms involving blood-flow-induced shear stress and adrenergic signaling [58]. In addition, the marked exercise-mediated suppression of tumor growth, could be attributed to the elevation of the levels of immune-attractant chemokines and NK cell-activating receptor ligands in the tumor microenviroment that associated with the increased epinephrine levels leads to mobilization of NK cells. Furthermore, another exercise-related physical factor, as increased body temperature can be also able to increase immune cell trafficking and function [59], since that hyperthermia can control and delay tumor growth through enhancement of intratumoral NK cell infiltration [60, 61]. These effects can be attributed to the fact that increased body temperature enhances immune cell trafficking by increasing the diameter of the intratumoral blood vessels. In addition to this physical effect, increased body temperature modifies the tumor vasculature by inducing IL-6 trans signaling, making the vasculature more permissible for cytotoxic T cell trafficking into the tumors [61].

An increase in the overall lymphocyte proliferation was observed in the melanoma-bearing animals on an exercise regimen in both the normolipidic [62] and high-fat groups (Fig. 4). This result was the opposite of that observed in the tumor-free animals, in which exercise significantly reduced the lymphocyte proliferation in the normolipidic group (N). In addition, lymphocyte production was significantly reduced in the sedentary high-fat diet-fed group (H) compared with that in the sedentary normolipidic (N) group, and the reduction in proliferation in the high-fat diet-fed group was not affected by the moderate exercise regimen (Fig. 4). In our study, differential effects of the high-fat diet and exercise regimen on various lymphocyte subsets (e.g., B vs. T; cytotoxic T vs. Treg) could account for the complex pattern of lymphocyte proliferation. One possibility is that the increase in lymphocyte proliferation with exercise in the tumor-bearing animals reflects an increased number of effector T lymphocytes, as T-cells reactive with tumor antigens undergo clonal expansion, but changes in other lymphocyte subpopulations contribute to the increased lymphocyte proliferation. Thus, future studies must be conducted to evaluate the effect of diet and exercise on specific T-cell types affecting tumor progression, because tumor growth is directly modulated by the function of CD4+ cells controlling tumor tolerance and by inflammatory cells augmenting tumor growth [63, 64].

In contrast with the results of proliferation, the release of IL-2 was decreased by physical exercise. It is worth to clarify that in the lymphocyte proliferation assay, cells were stimulated with ConA, a well-known mitogen agent, where the influence of IL-2 in T cell proliferation in this situation is minimized. In addition, it is also important to mention that in the assessment of cytokine production by lymphocytes in vitro, we used other agents as PMA and ionomicin. PMA activates protein kinase C, while ionomycin is a calcium ionophore; and stimulation with these compounds bypasses the T cell membrane receptor complex and leads to activation of several intracellular signaling pathways, resulting in T cell activation and production of a variety of cytokines, as well IL-2. Therefore, there are other important mediators that may influence T cell proliferation in the condition studied. In our model, we suppose that IL-2 is more involved with Th cell differentiation and less with proliferation. In fact, it has been demonstrated that exercise training increases lymphocyte proliferative response in cells from patients with chronic obstructive pulmonary disease [65]. One interesting study of Hutnick et al. [66] also showed that breast cancer patients submitted to moderate exercise following chemotherapy presented an elevated level of lymphocyte proliferation when cells were stimulated with ConA, without alterations of cytokine secretion. These results corroborate the findings reported in our study indicating that there are other factors modulated by physical exercise that may improve lymphocyte response in different tumor models.

Specific mechanisms that could transduce the observed effects of diet and/or exercise include T-cell suppression by inhibitors released by the tumor and the recruitment of T lymphocytes to the tumor site, which could inhibit actions of other leukocytes secreting pro-tumorigenic cytokines [67, 68]. Zaidi et al. [69] reported that IFN-gamma plays a crucial role in melanoma growth in neonatal mice exposed to UV radiation. In that study, the inhibition of IFN-gamma promoted a decrease in the inflammatory response associated with melanoma development. The decrease in lymphocyte IFN-gamma production promoted by physical exercise in obese mice suggests that blockage of this cytokine secretion caused by exercise plays a role in reducing tumor growth rate.

Tumor development is involved with some signaling pathways of the inflammatory response, such as the activation of transcription factors, including STAT3 and NF-kB, which are activated by growth factors [14]. IL-6, epithelial growth factor and vascular endothelial growth factor activate STAT3, thereby promoting increased expression of the anti-apoptotic Bcl-xL protein, which induces tumor cell survival, and preventing the synthesis of cytokines and growth factors that prevent the maturation of dendritic cells and decrease the recruitment capacity of CD8+ and NK cells.

Among the signaling pathways of inflammation that intersect between physical exercise training and the different stages of cancer, we can highlight the effects of physical exercise on the inflammatory response mediated by cytokines. The major cytokines already studied are IL-6 and IL10, IFN-alpha and gamma, and TNF-alpha. IL-6 may be increased by acute physical exercise but the intensity and duration may influence it concentration. However, IFN-gamma and TNF-alpha are decreased as a chronic adaptation to training [70, 71]. It is known that physical exercise activates the neuroendocrine system, releasing the corticotropin-releasing factor (CRH) and adrenaline by the sympathetic neurons and leading to the activation of the neuroendocrine system and the neuroendocrine axis [72]. The cytokines IL-10 and IL-4, produced by Th2 lymphocytes, are stimulated by glucocorticoids; this may be a form of protection against excessive production of proinflammatory cytokines [73]. This regulation corroborates the results observed in our study, which was characterized by the decrease in cytokine production by Th1 lymphocytes with inflammatory characteristics in the HEM animals in relation to the HM animals, evidencing a possible action of exercise on the modulation of the immunoinflammatory and neuroendocrine axes.

It is essential to highlight that this is the first study to investigate the effect of moderate physical exercise on tumor non-infiltrated lymphocytes in melanoma condition. The investigation of these cells are extremely important in clinical aspects due to the fact that understanding mechanisms to promote improvement in the lymphocyte function may lead to a better response to cancer treatments, including chemotherapy as demonstrated by Hutnick et al. [66].

## Conclusions

**Fig. 7:**
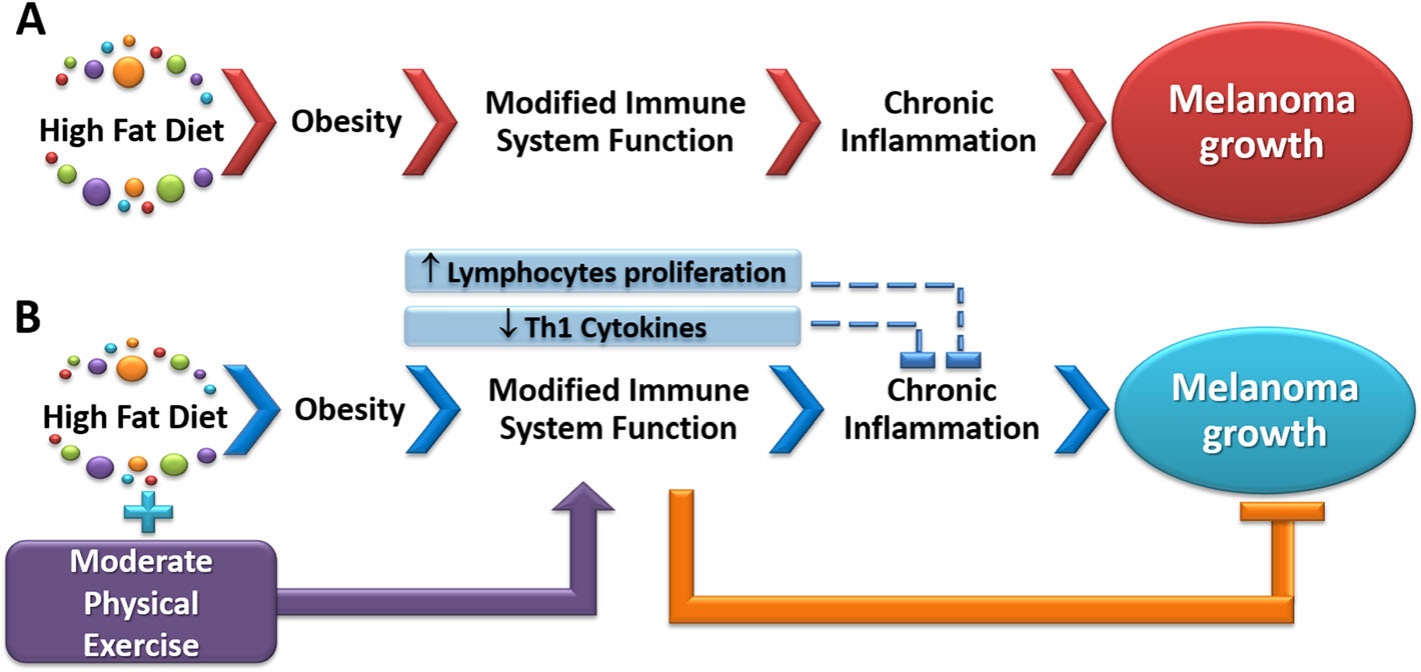
Representative scheme of the results. (**a**) Results observed in the animals fed the high-fat diet + melanoma (HM). (**b**) Results of the association between the high-fat diet and physical exercise

In conclusion, a high-fat diet not only induced obesity but also favored melanoma growth. When the high-fat diet-fed animals were concomitantly submitted to a regimen of moderate physical exercise, melanoma growth was significantly reduced. Other important changes with exercise in high-fat diet-fed animals bearing tumors were increased and tumor non-infiltrated lymphocytes proliferation, decreased serum levels of leptin, and decreased production of cytokines involved in the Th1 response, which would suggest a decrease in chronic inflammation that accompanies both obesity and tumor development. The decreased Th1 response might play a major role in decreasing tumor volume and tumor weight. The results of the present study support the hypothesis that altering function of tumor non-infiltrated lymphocytes via exercise-related mechanisms can slow melanoma progression, indicating that the incorporation of a regular practice of moderate-intensity exercises can be a potential strategy for current therapeutic regimens in treating advanced melanoma. A summary of the results is reported in Fig. 7.

## Data Availability

The datasets used during the present study are available from the corresponding author upon request.

## Abbreviations

Bcl-xL: B-cell lymphoma-extra-large
BrdU: Bromodeoxyuridine
BSA: Bovine serum albumin
CRH: Corticotropin-releasing factor
EDTA: Ethylene-Diamine-Tetra-Acetic acid
FBS: Fetal bovine serum
H: High-fat control
HE: High-fat control + physical exercise
HEM: High-fat melanoma + physical exercise
HM: High-fat + melanoma
IL: Interleukin
N: Normolipidic control
NE: Normolipidic control + physical exercise
NEM: Normolipidic melanoma + physical exercise
NF: Nuclear factor
NK: Natural killer cells
NM: Normolipidic + melanoma
PBS: Phosphate-buffered saline
PD-1: Cell death protein 1
PMA: Phorbol myristate acetate
ROS: Reactive oxygen species
STAT: Signal transducers and activators of transcription
Th: T helper cell
TNF: Tumor necrosis factor
Treg: T-regulatory cell

## References

[1] Friedenreich CM, Orenstein MR (2002). Physical activity and cancer prevention: etiologic evidence and biological mechanisms. J Nutr.

[2] Greenwald P, Clifford CK, Milner JA (2001). Diet and cancer prevention. Eur J Cancer.

[3] Faggioni R, Feingold KR, Grunfeld C (2001). Leptin regulation of the immune response and the immunodeficiency of malnutrition. FASEB J.

[4] Gogas H, Trakatelli M, Dessypris N, Terzidis A, Katsambas A, Chrousos GP, Petridou ET (2008). Melanoma risk in association with serum leptin levels and lifestyle parameters: a case-control study. Ann Oncol.

[5] Oda A, Taniguchi T, Yokoyama M (2001). Leptin stimulates rat aortic smooth muscle cell proliferation and migration. Kobe J Med Sci.

[6] Galgani Mario, Procaccini Claudio, De Rosa Veronica, Carbone Fortunata, Chieffi Paolo, La Cava Antonio, Matarese Giuseppe (2010). Leptin Modulates the Survival of Autoreactive CD4+ T Cells through the Nutrient/Energy-Sensing Mammalian Target of Rapamycin Signaling Pathway. The Journal of Immunology.

[7] Lambert Joshua D., Elias Ryan J. (2010). The antioxidant and pro-oxidant activities of green tea polyphenols: A role in cancer prevention. Archives of Biochemistry and Biophysics.

[8] de Aquino MT, Malhotra A, Mishra MK, Shanker A. Challenges and future perspectives of T cell immunotherapy in cancer. Immunol Lett. 166:117–33. 10.1016/j.imlet.2015.05.018 PMC4499494 26096822

[9] Ghigliotti Giorgio, Barisione Chiara, Garibaldi Silvano, Fabbi Patrizia, Brunelli Claudio, Spallarossa Paolo, Altieri Paola, Rosa Gianmarco, Spinella Giovanni, Palombo Domenico, Arsenescu Razvan, Arsenescu Violeta (2014). Adipose Tissue Immune Response: Novel Triggers and Consequences for Chronic Inflammatory Conditions. Inflammation.

[10] Bilski J, Brzozowski B, Mazur-Bialy A, Sliwowski Z, Brzozowski T. The role of physical exercise in inflammatory bowel disease. Biomed Res Int. 2014;429031. 10.1155/2014/429031 PMC4022156 24877092

[11] Davis HR, Mullins DE, Pines JM, Hoos LM, France CF, Compton DS, Graziano MP, Sybertz EJ, Strader CD, Van Heek M (1998). Effect of chronic central administration of glucagon-like peptide-1 (7-36) amide on food consumption and body weight in normal and obese rats. Obes Res.

[12] Jonsdottir IH, Hoffmann P (2000). The significance of intensity and duration of exercise on natural immunity in rats. Med Sci Sports Exerc.

[13] Curran M. A., Montalvo W., Yagita H., Allison J. P. (2010). PD-1 and CTLA-4 combination blockade expands infiltrating T cells and reduces regulatory T and myeloid cells within B16 melanoma tumors. Proceedings of the National Academy of Sciences.

[14] Berasain C, Castillo J, Perugorria MJ, Latasa MU, Prieto J, Avila MA (2009). Inflammation and liver cancer: new molecular links. Ann N Y Acad Sci.

[15] Chen CH, Lee HS, Huang GT, Yang PM, Yu WY, Cheng KC, Lee PH, Jeng YM, Chen DS, Sheu JC (2007). Phenotypic analysis of tumor-infiltrating lymphocytes in hepatocellular carcinoma. Hepatogastroenterology.

[16] Speretta Guilherme F., Silva André A., Vendramini Regina C., Zanesco Angelina, Delbin Maria A., Menani José V., Bassi Mirian, Colombari Eduardo, Colombari Débora S.A. (2016). Resistance training prevents the cardiovascular changes caused by high-fat diet. Life Sciences.

[17] Pereira B, Rosa LF, Safi DA, Bechara EJ, Curi R (1995). Hormonal regulation of superoxide dismutase, catalase, and glutathione peroxidase activities in rat macrophages. Biochem Pharmacol.

[18] Bacurau RF, Belmonte MA, Seelaender MC, Costa Rosa LF (2000). Effect of a moderate intensity exercise training protocol on the metabolism of macrophages and lymphocytes of tumour-bearing rats. Cell Biochem Funct.

[19] Momesso dos Santos CM, Sato FT, Cury-Boaventura MF, Guirado-Rodrigues SH, Cacula KG, Goncalves Santos CC, Hatanaka E, de Oliveira HH, Santos VC, Murata G (2015). Effect of regular circus physical exercises on lymphocytes in overweight children. PLoS One.

[20] SEER Cancer Statistics Factsheets**:** Melanoma of the Skin. [http://seer.cancer.gov/statfacts/html/melan.html].

[21] Brandner Johanna M., Haass Nikolas K. (2013). Melanoma’s connections to the tumour microenvironment. Pathology.

[22] Mantovani A (2009). The yin-yang of tumor-associated neutrophils. Cancer Cell.

[23] Hanahan Douglas, Weinberg Robert A. (2011). Hallmarks of Cancer: The Next Generation. Cell.

[24] Kranendonk ME, van Herwaarden JA, Stupkova T, de Jager W, Vink A, Moll FL, Kalkhoven E, Visseren FL (2015). Inflammatory characteristics of distinct abdominal adipose tissue depots relate differently to metabolic risk factors for cardiovascular disease: distinct fat depots and vascular risk factors. Atherosclerosis.

[25] Voltarelli FA, Frajacomo FT, Padilha CS, Testa MTJ, Cella PS, Ribeiro DF, de Oliveira DX, Veronez LC, Bisson GS, Moura FA, Deminice R (2017). Syngeneic B16F10 melanoma causes Cachexia and impaired skeletal muscle strength and locomotor activity in mice. Front Physiol.

[26] Bachi AL, Dos Santos LC, Nonogaki S, Jancar S, Jasiulionis MG (2012). Apoptotic cells contribute to melanoma progression and this effect is partially mediated by the platelet-activating factor receptor. Mediat Inflamm.

[27] Bachi AL, Kim FJ, Nonogaki S, Carneiro CR, Lopes JD, Jasiulionis MG, Correa M (2009). Leukotriene B4 creates a favorable microenvironment for murine melanoma growth. Mol Cancer Res.

[28] Correa M, Machado J, Carneiro CR, Pesquero JB, Bader M, Travassos LR, Chammas R, Jasiulionis MG (2005). Transient inflammatory response induced by apoptotic cells is an important mediator of melanoma cell engraftment and growth. Int J Cancer.

[29] Jaber DF, Jallad MN, Abdelnoor AM (2017). The effect of ciprofloxacin on the growth of B16F10 melanoma cells. J Cancer Res Ther.

[30] Contreras A, Sen S, Tatar AJ, Mahvi DA, Meyers JV, Srinand P, Suresh M, Cho CS (2016). Enhanced local and systemic anti-melanoma CD8+ T cell responses after memory T cell-based adoptive immunotherapy in mice. Cancer Immunol Immunother.

[31] Hammond-McKibben D, Lake P, Zhang J, Tart-Risher N, Hugo R, Weetall M (2001). A high-capacity quantitative mouse model of drug-mediated immunosuppression based on rejection of an allogeneic subcutaneous tumor. J Pharmacol Exp Ther.

[32] Ferreira JC, Rolim NP, Bartholomeu JB, Gobatto CA, Kokubun E, Brum PC (2007). Maximal lactate steady state in running mice: effect of exercise training. Clin Exp Pharmacol Physiol.

[33] Mehl KA, Davis JM, Clements JM, Berger FG, Pena MM, Carson JA (2005). Decreased intestinal polyp multiplicity is related to exercise mode and gender in ApcMin/+ mice. J Appl Physiol (1985).

[34] Evans ES, Hackney AC, McMurray RG, Randell SH, Muss HB, Deal AM, Battaglini CL (2015). Impact of acute intermittent exercise on natural killer cells in breast Cancer survivors. Integr Cancer Ther.

[35] Rogers P, Webb GP (1980). Estimation of body fat in normal and obese mice. Br J Nutr.

[36] Alp PR, Newsholme EA, Zammit VA (1976). Activities of citrate synthase and NAD+-linked and NADP+-linked isocitrate dehydrogenase in muscle from vertebrates and invertebrates. Biochem J.

[37] Ardawi MS, Newsholme EA (1982). Maximum activities of some enzymes of glycolysis, the tricarboxylic acid cycle and ketone-body and glutamine utilization pathways in lymphocytes of the rat. Biochem J.

[38] ZHAO Li, QIU De Kai, MA Xiong (2010). Th17 cells: The emerging reciprocal partner of regulatory T cells in the liver. Journal of Digestive Diseases.

[39] Guery L, Hugues S. Th17 cell plasticity and functions in Cancer immunity. Biomed Res Int. 2015;314620. 10.1155/2015/314620 PMC4637016 26583099

[40] Berger S, Ceccarini G, Scabia G, Barone I, Pelosini C, Ferrari F, Magno S, Dattilo A, Chiovato L, Vitti P, et al. Lipodystrophy and obesity are associated with decreased number of T cells with regulatory function and pro-inflammatory macrophage phenotype. Int J Obes. 2017. 10.1038/ijo.2017.163 28761130

[41] Papathanassoglou E, El-Haschimi K, Li XC, Matarese G, Strom T, Mantzoros C (2006). Leptin receptor expression and signaling in lymphocytes: kinetics during lymphocyte activation, role in lymphocyte survival, and response to high fat diet in mice. J Immunol.

[42] Pedersen L, Idorn M, Olofsson GH, Lauenborg B, Nookaew I, Hansen RH, Johannesen HH, Becker JC, Pedersen KS, Dethlefsen C (2016). Voluntary running suppresses tumor growth through epinephrine- and IL-6-dependent NK cell mobilization and redistribution. Cell Metab.

[43] McQuade JL, Daniel CR, Hess KR, Mak C, Wang DY, Rai RR, Park JJ, Haydu LE, Spencer C, Wongchenko M (2018). Association of body-mass index and outcomes in patients with metastatic melanoma treated with targeted therapy, immunotherapy, or chemotherapy: a retrospective, multicohort analysis. Lancet Oncol.

[44] Jones LW, Viglianti BL, Tashjian JA, Kothadia SM, Keir ST, Freedland SJ, Potter MQ, Moon EJ, Schroeder T, Herndon JE, Dewhirst MW (1985). Effect of aerobic exercise on tumor physiology in an animal model of human breast cancer. J Appl Physiol.

[45] Chen GL, Luo Y, Eriksson D, Meng X, Qian C, Bauerle T, Chen XX, Schett G, Bozec A (2016). High fat diet increases melanoma cell growth in the bone marrow by inducing osteopontin and interleukin 6. Oncotarget.

[46] Nieman KM, Romero IL, Van Houten B, Lengyel E (1831). Adipose tissue and adipocytes support tumorigenesis and metastasis. Biochim Biophys Acta.

[47] Robado de Lope L, Alcibar OL, Amor Lopez A, Hergueta-Redondo M, Peinado H. Tumour-adipose tissue crosstalk: fuelling tumour metastasis by extracellular vesicles. Philos Trans R Soc Lond Ser B Biol Sci. 2018:373. 10.1098/rstb.2016.0485 PMC5717439 29158314

[48] McCullough DJ, Nguyen LM, Siemann DW, Behnke BJ (2013). Effects of exercise training on tumor hypoxia and vascular function in the rodent preclinical orthotopic prostate cancer model. J Appl Physiol (1985).

[49] Pandit R, Beerens S, Adan RAH (2017). Role of leptin in energy expenditure: the hypothalamic perspective. Am J Physiol Regul Integr Comp Physiol.

[50] Lord GM, Matarese G, Howard JK, Baker RJ, Bloom SR, Lechler RI (1998). Leptin modulates the T-cell immune response and reverses starvation-induced immunosuppression. Nature.

[51] Chopra M, Riedel SS, Biehl M, Krieger S, von Krosigk V, Bauerlein CA, Brede C, Jordan Garrote AL, Kraus S, Schafer V (2013). Tumor necrosis factor receptor 2-dependent homeostasis of regulatory T cells as a player in TNF-induced experimental metastasis. Carcinogenesis.

[52] Matarese G, Procaccini C, De Rosa V, Horvath TL, La Cava A (2010). Regulatory T cells in obesity: the leptin connection. Trends Mol Med.

[53] Mesquita D, Cruvinel WM, Camara NO, Kallas EG, Andrade LE (2009). Autoimmune diseases in the TH17 era. Braz J Med Biol Res.

[54] Kawanishi N, Yano H, Yokogawa Y, Suzuki K. Exercise training inhibits inflammation in adipose tissue via both suppression of macrophage infiltration and acceleration of phenotypic switching from M1 to M2 macrophages in high-fat-diet-induced obese mice. Exerc Immunol Rev. 16:105–18. 20839495

[55] Jovicic N, Jeftic I, Jovanovic I, Radosavljevic G, Arsenijevic N, Lukic ML, Pejnovic N (2015). Differential Immunometabolic phenotype in Th1 and Th2 dominant mouse strains in response to high-fat feeding. PLoS One.

[56] Dunn GP, Old LJ, Schreiber RD (2004). The three Es of cancer immunoediting. Annu Rev Immunol.

[57] Hojman P (2017). Exercise protects from cancer through regulation of immune function and inflammation. Biochem Soc Trans.

[58] Idorn M, Hojman P (2016). Exercise-dependent regulation of NK cells in Cancer protection. Trends Mol Med.

[59] Evans SS, Repasky EA, Fisher DT (2015). Fever and the thermal regulation of immunity: the immune system feels the heat. Nat Rev Immunol.

[60] Chen P, Yang LL, Yang HS, Wang YS, Li G, Wu Y, Fang F, Liu K, Li J, Zhao X (2008). Synergistic antitumor effect of CXCL10 with hyperthermia. J Cancer Res Clin Oncol.

[61] Fisher DT, Chen Q, Skitzki JJ, Muhitch JB, Zhou L, Appenheimer MM, Vardam TD, Weis EL, Passanese J, Wang WC (2011). IL-6 trans-signaling licenses mouse and human tumor microvascular gateways for trafficking of cytotoxic T cells. J Clin Invest.

[62] Wan X, Guloglu FB, VanMorlan AM, Rowland LM, Jain R, Haymaker CL, Cascio JA, Dhakal M, Hoeman CM, Tartar DM, Zaghouani H (2012). Mechanisms underlying antigen-specific tolerance of stable and convertible Th17 cells during suppression of autoimmune diabetes. Diabetes.

[63] Rhee I (2016). Diverse macrophages polarization in tumor microenvironment. Arch Pharm Res.

[64] Zaidi MR, Merlino G (2011). The two faces of interferon-gamma in cancer. Clin Cancer Res.

[65] Fernandes JR, da Silva CCB M, da Silva AG, de Carvalho Pinto RM, da Silva Duarte AJ, Carvalho CR, Benard G (2018). Effect of an exercise program on lymphocyte proliferative responses of COPD patients. Lung.

[66] Hutnick NA, Williams NI, Kraemer WJ, Orsega-Smith E, Dixon RH, Bleznak AD, Mastro AM (2005). Exercise and lymphocyte activation following chemotherapy for breast cancer. Med Sci Sports Exerc.

[67] Shimizu J, Yamazaki S, Sakaguchi S (1999). Induction of tumor immunity by removing CD25+CD4+ T cells: a common basis between tumor immunity and autoimmunity. J Immunol.

[68] Szczepanski MJ, Czystowska M, Szajnik M, Harasymczuk M, Boyiadzis M, Kruk-Zagajewska A, Szyfter W, Zeromski J, Whiteside TL (2009). Triggering of toll-like receptor 4 expressed on human head and neck squamous cell carcinoma promotes tumor development and protects the tumor from immune attack. Cancer Res.

[69] Zaidi MR, Davis S, Noonan FP, Graff-Cherry C, Hawley TS, Walker RL, Feigenbaum L, Fuchs E, Lyakh L, Young HA, et al. Interferon-gamma links ultraviolet radiation to melanomagenesis in mice. Nature. 469:548–53. 10.1038/nature09666 PMC3140101 21248750

[70] Farinha JB, Steckling FM, Stefanello ST, Cardoso MS, Nunes LS, Barcelos RP, Duarte T, Kretzmann NA, Mota CB, Bresciani G (2015). Response of oxidative stress and inflammatory biomarkers to a 12-week aerobic exercise training in women with metabolic syndrome. Sports Med Open.

[71] Reihmane D, Dela F (2014). Interleukin-6: possible biological roles during exercise. Eur J Sport Sci.

[72] Pedersen BK, Bruunsgaard H, Klokker M, Kappel M, MacLean DA, Nielsen HB, Rohde T, Ullum H, Zacho M (1997). Exercise-induced immunomodulation--possible roles of neuroendocrine and metabolic factors. Int J Sports Med.

[73] Greiwe JS, Cheng B, Rubin DC, Yarasheski KE, Semenkovich CF (2001). Resistance exercise decreases skeletal muscle tumor necrosis factor alpha in frail elderly humans. FASEB J.

